# Is Italian Dentists’ Knowledge of Enamel Development Defects Adequate? A Nationwide Survey

**DOI:** 10.1016/j.identj.2024.04.013

**Published:** 2024-04-28

**Authors:** Claudia Salerno, Guglielmo Campus, Nicole Camoni, Silvia Cirio, Alberto Caprioglio, Maria Grazia Cagetti

**Affiliations:** a Department of Restorative, Preventive and Pediatric Dentistry, University of Bern, Bern, Switzerland; b Department of Biomedical, Surgical and Dental Sciences, University of Milan, Milan, Italy; c Graduate School for Health Sciences, University of Bern, Switzerland; d Department of Medicine, Surgery and Pharmacy, University of Sassari, Sassari, Italy; e Department of Cariology, Saveetha Dental College and Hospitals, Chennai, Tamil Nadu, India; f Department of Biomedical, Surgical and Dental Sciences, School of Dentistry, University of Milan, Milan, Italy; g Fondazione IRCCS Cà Granda, Ospedale Maggiore Policlinico, Milan, Italy

**Keywords:** Developmental defects of enamel, Molar incisor hypomineralisation, Amelogenesis imperfecta, Dental fluorosis, Dentists’ knowledge

## Abstract

**Objectives:**

Correct identification and management of Developmental Defects of Enamel (DDEs) are essential to provide the best possible treatment. The present survey aims to investigate Italian dentists’ knowledge of DDEs, their ability to recognise the different clinical pictures, and to choose the most appropriate clinical approach.

**Methods:**

A cross-sectional survey was planned based on a questionnaire including 27 closed-ended questions, and that proposed 4 clinical pictures, molar incisor hypomineralisation (MIH), amelogenesis imperfecta (AI), dental fluorosis (DF), and an initial caries lesion (ICL). It was distributed by e-mail to all Italian dentists (N = 63,883) through the Italian Federation of Doctors and Dentists. Discrete variables were expressed as absolute and relative frequencies (%). A multivariate analysis assessed whether socio-demographic variables correlated with the answers’ truthfulness.

**Results:**

About 5017 questionnaires were included and analysed. Although 90.19% of the sample stated that they had received information on DDEs, a significant percentage did not recognise MIH (36.36%), AI (48.34%), DF (71.50%), and ICL (46.62%). Only 57.07% correctly classified enamel hypomineralisation as a qualitative defect, and even fewer, 54.45%, classified enamel hypoplasia as a quantitative defect. According to the logistic regressions, female dentists, dentists who treat mainly children and received information about DDEs, were more likely to recognise the 4 clinical pictures (*P* < .01).

**Conclusions:**

Italian dentists showed many knowledge gaps on DDEs that need to be filled; those who received formal training were more capable of correctly identifying the defects and were more likely to prescribe an appropriate management approach for the defects.

**Clinical significance:**

Increasing university courses and continuing education on diagnosing and managing DDEs seems reasonable to fill the knowledge gap on DDEs.

## Introduction

Developmental Defects of Enamel (DDEs) are a heterogeneous set of structural abnormalities of varying severity that can occur during the formation and mineralisation of dental enamel.^1^ These defects can be attributed to genetic, environmental, or systemic factors and arise at different stages of enamel deposition and maturation.^2^ Understanding the aetiology, common symptoms, classifications, and treatment modalities of DDEs is vital for dental practitioners, as these defects impact the aesthetic appearance of teeth and can create challenges to oral health.^3^

DDEs are becoming increasingly common worldwide, although it can be difficult to accurately assess how common they are due to the high incidence of dental caries on the affected enamel, which can mask the original defect.^4^ The defects may impact the primary and permanent dentition, may be limited to one or more teeth, and exhibit a broad range of sizes, colours, and shapes.^5^ The enamel texture of the affected teeth can be significantly altered; the defects may be either qualitative (hypomineralisation, manifested as white, yellow, or brown opacities) or quantitative (hypoplasia, manifested as pits, grooves, or a more severe deficit) or a combination of the 2. The stage of amelogenesis, mainly in utero and early childhood, at which the pathogenic factor acts determines the type and severity of the anomaly: if it acts in the secretory phase, hypoplasia occurs; if it acts in the maturation phase, hypomineralisation occurs.^4^ An accurate estimation of the approximate period of the injury may be obtained with a solid understanding of the history of tooth development.^6^ Genetic susceptibility, environmental exposures, and systemic disturbances can interfere with the complex process of amelogenesis, leading to abnormalities in enamel structure. Factors such as malnutrition, prenatal exposure to toxins, childhood illnesses, and certain medications can play a role into the occurrence of DDEs.^7^, ^8^, ^9^

Due to the enamel's integrity being compromised by DDEs, masticatory function, tooth/teeth sensitivity, or aesthetics may be negatively impacted. These elements may lead to poor oral hygiene or tooth fracture (post-eruptive breakdown), which increase the risk of dental caries in the affected teeth.^10^

Accurate diagnosis of DDEs is mandatory for efficient treatment planning and preventive measures. Early intervention can minimise the defects’ progression and avoid subsequent consequences. In addition, accurate diagnosis helps to address any underlying systemic problems, thus promoting comprehensive health care.

There are multiple systems to classify the diverse types of DDEs. The Fédération Dentaire Internationale (FDI) recommends the use of the Developmental Defects of Enamel (DDE) Index, which includes factors such as type (opacity, hypoplasia, discoloration), number (single or multiple), demarcation (demarcated or diffuse), and the location of the defects on the buccal and lingual surfaces of the teeth.^11^ Understanding these classifications aids in standardising diagnoses and facilitating communication among dental professionals, researchers, and clinicians.

The management of DDEs requires a multi-disciplinary assessment of both aesthetic and functional aspects. Treatment options depend on the severity, defect type, and tooth affected. Mild cases can be handled with non-invasive or minimally invasive techniques, such as high-concentration fluoride products, resin infiltration, or direct restorations, while more severe cases may require crowns, veneers, or other extensive restorative procedures.^12^, ^13^, ^14^, ^15^, ^16^ The adhesion of composite resins to the affected enamel is often ineffective, making the prognosis poor in the medium to long term.^17^ Subjects with DDEs may also exhibit significant levels of dental anxiety due to severe tooth hypersensitivity, which makes managing them challenging. The choice of treatment is often tailored to the individual needs and preferences of the patient.

Against this background, it is possible to deduce how DDEs represent a multifaceted challenge in paediatric and general dentistry. A correct approach to the diagnosis and management of DDEs is essential to ensure optimal oral health outcomes and improve the quality of life of those affected. Still, one question arises: are dentists adequately trained on the subject? Data on dentists’ knowledge, diagnostic skills, and therapeutic choice on DDEs have not yet been presented worldwide; only one survey assessed these aspects of DDEs of undergraduate dental and hygiene students in Italy.^18^ Few surveys have studied dentists’ knowledge of individual enamel defects such as MIH, not investigating the topic in its entirety.^19^, ^20^, ^21^, ^22^, ^23^, ^24^

Based on these premises, the present paper aims to investigate Italian dentists’ knowledge of developmental defects of enamel, their ability to recognise the different clinical pictures, and their choice of the more appropriate clinical approach. A nationwide anonymous questionnaire was adapted and distributed to achieve this goal.

## Materials and methods

The study was designed as an observational, questionnaire-based, cross-sectional study; it complied with the Declaration of Helsinki and was performed according to ethics committee approval (Ethics Committee Board of Sassari University, Sassari, Italy, N°AOU_SS 97 on 11 November 2021). The reporting of this study follows the Standards for Reporting of Diagnostic Accuracy guidelines.^25^

The questionnaire was adapted based on a previously validated Italian questionnaire.^18^ It consisted of 27 closed-ended questions in dichotomous, multiple-choice, or Likert scales (Supplementary file S1). Only the questions on demographic characteristics were adapted to the sample, as the questionnaire would be administered to graduating dentists and not to students. A quantitative analysis of the accuracy of the questionnaire was performed by submitting it to 12 experts (5 dentists specialised in Paediatric Dentistry with more than 5 years of experience, 4 academics, and 3 clinical researchers). The quantitative content validity of each item was assessed using the Content Validity Index (CVI) and the Content Validity Ratio (CVR).^26^ The Scale Content Validity Index (S-CVI) was finally calculated using the universal agreement method. Based on experts’ opinions, the S-CVI and S-CVR for the entire tool were 1.00 and 0.99, respectively (Supplementary file S2).

The final version was pre-tested in September 2021 for comprehensibility on a small sample of 30 general dentists not included in the survey. After completing the questionnaire, they were contacted to find out if they had experienced any difficulty in understanding the questions and were given a comprehension score from 1 (extreme difficulty) to 5 (no difficulty). A result of 4.47±0.12 was obtained.

The questionnaire includes 4 questions exploring the demographic characteristics of the sample, 11 questions investigating the basic knowledge acquired about DDEs, and 12 questions verifying the ability of dentists to distinguish among different DDEs through the presentation of 4 clinical images (Figure).

**Figure:**
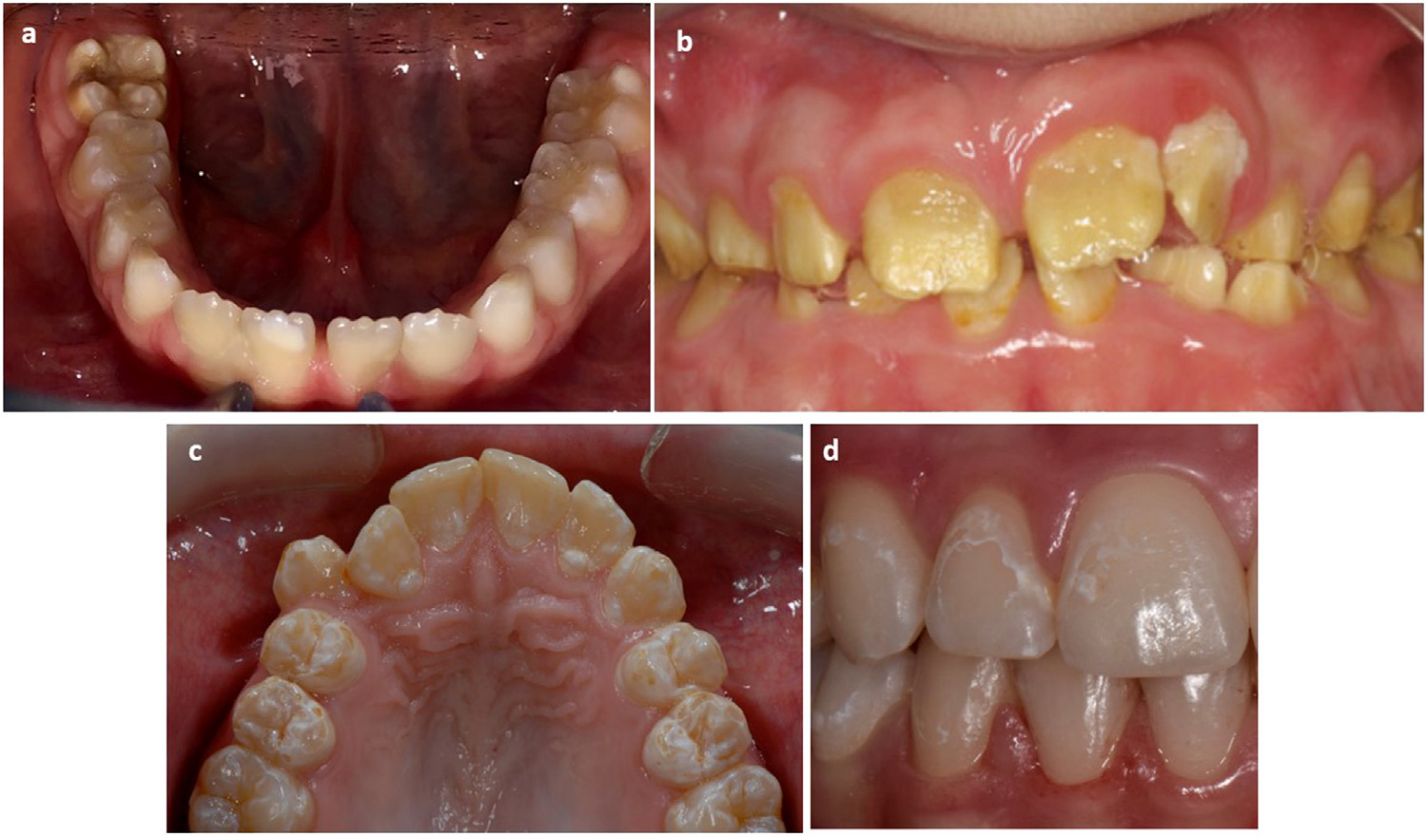
Clinical pictures shown in the questionnaire: a, molar incisor hypomineralisation; b, amelogenesis imperfecta; c, dental fluorosis; d, initial caries lesion (ICL).

The socio-demographic data required were gender, age, years of working experience, type of patient predominantly treated, and whether and where knowledge of DDEs was acquired. The first clinical image showed first molars affected by severe molar incisor hypomineralisation (MIH), the second image showed an entire permanent dentition affected by amelogenesis imperfecta (AI) (hypo-mineralised type), the third image showed upper canines and premolars affected by Dental Fluorosis (moderate grade, following Dean classification^27^) and the last image showed an initial caries lesion (ICL) (ICDAS score of 2).^28^ After recognising each defect, the participant was asked to express an opinion on each defect's caries risk (as low, medium, or high) and to choose the treatment they considered appropriate.

An online version of the anonymous questionnaire was distributed via email. Italian dentists were contacted using email addresses from the Italian Federation of Medical Doctors and Dentists, as all dentists licensed in Italy must provide an email address. No follow-ups or re-invitations were issued to non-responders. A description of the purpose of the study was also included before the first question, and dentists were asked to sign an online informed consent form under the Italian data protection law. If they did not sign the consent, the questionnaire was automatically closed.

The survey was conducted from January 2022 until December 2022. Data were collected in January 2023.

A priori power analysis was used to calculate the sample size. Given the national population of dentists of 63,883,^29^ the minimum sample size resulted in 1480 dentists with an anticipated frequency of 50%, a power of 99.99%, a design effect of 1, and an alfa error at 0.05.

### Statistical analysis

The deidentified data were downloaded from the survey site, imported into a Microsoft Excel spreadsheet, and quality-checked by an author to ensure accuracy. Data from participants whose questionnaires were incomplete or whose responses to the sentinel question were inconsistent were excluded. Only dentists with an Italian degree in Dentistry were enrolled. Descriptive statistics were calculated for all items to provide an overview of the results. Analyses were conducted using STATA 18.0 SE. Discrete variables were expressed as absolute and relative frequencies (%). The alpha risk was set to 5%.

Multivariate analysis was performed to assess whether sociodemographic variables were related to the truthfulness of the response. For logistic regression models (STATA's logit command), sociodemographic variables, caries risk classification, and the type of suitable therapy were used as the explanatory variable, and the correct response related to the framing of each clinical image as dependent variables. For multivariate analysis, categorical variables were re-coded in numerical variables (Supplementary file S3). The data were checked for multicollinearity using the Belsley–Kuh–Welsch technique. The heteroscedasticity and normality of the residuals were assessed using the White test and the Shapiro–Wilk test, respectively. The interaction model (likelihood ratio test statistic) evaluated potential effects modifiers.

## Results

Of all Italian dentists, 6298 dentists opened the questionnaire with a response rate of 9.86%; 18 dentists (0.28%) did not sign the informed consent, and 1263 dentists (20.05%) did not complete or answer the questionnaire properly (inconsistent sentinel questions). Therefore, questionnaires filled by 5017 dentists were included and analysed with a final response rate of 7.85%.

The study sample included 59.70% of males, with diverse age distributions. Regarding work experience, 59.88% had over 16 years of experience. Professional focus showed 81.66% primarily working with adults. Concerning knowledge of DDEs, 90.19% received information. The primary sources were university lectures (68.27%), conferences or non-university courses (35.18%), books (40.64%), and the Internet (16.54%). Only 9.81% had never received information on DDEs (Table 1).

**Table 1:** Demographic characteristics of the sample.

	Total = 5017
Item	N (%)
**Gender**	
Male	2995 (59.70)
Female	2022 (40.30)
**Age (years)**	
≤30	491 (9.79)
31-40	1146 (22.84)
41-50	1164 (23.20)
51-60	1082 (21.57)
≥ 60	1134 (22.60)
**How many years have you been practicing?**	
1-5	774 (15.43)
6-10	574 (11.44)
11-15	665 (13.25)
≥ 16	3004 (59.88)
**Which patients do you mainly treat?**	
Children (≤14 years)	796 (15.87)
Adults	4097 (81.66)
Olders (≥65 years)	124 (2.47)
**Have you received information regarding DDEs?**	
No	492 (9.81)
Yes	4525 (90.19)
**If yes, where?**	
University lessons	3425 (68.27)
Congresses or extra-university courses	1765 (35.18)
Books	2039 (40.64)
Internet	830 (16.54)
Other sources	148 (2.95)

Only 57.07% correctly classified enamel hypomineralisation as a qualitative defect, and even fewer, 54.45%, classified enamel hypoplasia as a quantitative defect. Just over one-third of the participants (39.90%) recognised MIH as a qualitative defect. Still, a higher percentage (67.49%) were aware that MIH develops in the pre-eruptive phase, with 66.12% recognising its multifactorial aetiology. Regarding amelogenesis imperfecta, the majority (86.33%) recognised its development in the pre-eruptive phase, but only 45.03% knew it had a genetic aetiology. Less than half (44.47%) were aware that dental fluorosis occurs in the pre-eruptive phase, and almost half (46.42%) believed that it could be confused with an ICL (Table 2).

**Table 2:** Dentists’ basic knowledge acquired on DDEs.

	Total = 5017
Item	N (%)
**Enamel hypomineralisation is a …… defect:**	
Qualitative	2863 (57.07)
Quantitative/Both/I don't know	2154 (42.93)
**Enamel hypoplasia is a ……. defect:**	
Quantitative	2732 (54.45)
Qualitative/Both/I don't know	2285(45.55)
**Molar incisor hypomineralisation (MIH) is a …… defect:**	
Qualitative	2002 (39.90)
Quantitative/Both/I don't know	3015 (60.10)
**When does molar incisor hypomineralisation develop?**	
In the pre-eruptive phase	3386 (67.49)
In the post-eruptive phase/any age/I don't know	1631 (32.51)
**Molar incisor hypomineralisation (MIH) is a condition caused by:**	
Multifactorial	3317 (66.12)
Genetic factors/Systemic factors/viral or bacterial infection/ I don't know	1700 (33.89)
**When does amelogenesis imperfecta develop?**	
In the pre-eruptive phase	4331 (86.33)
In the post-eruptive phase/any age/I don't know	686 (13.67)
**Amelogenesis imperfecta is a condition caused by:**	
Genetic factors	2259 (45.03)
Systemic factors/multifactorial/viral or bacterial infection/ I don't know	2758 (54.97)
**When does dental fluorosis develop?**	
In the pre-eruptive phase	2231 (44.47)
In the post-eruptive phase/any age/I don't know	2786 (55.53)
**Do you think fluorosis can be confused with plaque demineralisation (initial caries lesion)?**	
Yes	2329 (46,42)
No	2070 (41,26)
I don't know	618 (12,32)

A significant proportion of the dentists interviewed did not recognise the clinical images; MIH was identified by 36.36%, amelogenesis imperfecta by 51.66%, dental fluorosis by 28.50%, and the ICL by 53.38%. Most participants considered all teeth shown to be at high risk of caries. The most frequently recommended treatments included fluoride-based remineralising products, restorative therapy, and resin-based sealants (Table 3).

**Table 3:** Dentists’ ability to recognise 4 different clinical pictures, the caries risk, and the most appropriate clinical approach chosen.

	Total = 5017
Items	N (%)
**Picture 1: molar incisor hypomineralisation**	
Molar incisor hypomineralisation (MIH)	1824 (36.36)
Amelogenesis imperfecta	1473 (29.36)
Dental fluorosis	761 (15.17)
Initial caries lesion	292 (5.82)
Other	269 (5.36)
I don't know	398 (7.93)
**The caries risk in this situation is generally:**	
High	3200 (63.78)
Medium	1506 (30.02)
Low	311 (6.20)
**Which of the following treatments would you recommend?**	
Remineralising products and/or fluoride-based varnish or gel	3653 (72.81)
Glass-ionomer sealants	1549 (30.88)
Resin-based sealants	2161 (43.07)
Professional bleaching	482 (9.61)
Restorative treatment	2979 (59.38)
**Picture 2: amelogenesis imperfecta**	
Amelogenesis imperfecta	2592 (51.66)
Initial caries lesions	923 (18.40)
Dental fluorosis	614 (12.24)
Molar incisor hypomineralisation (MIH)	213 (4.25)
Other	181 (3.61)
I don't know	494 (9.85)
**The caries risk in this situation is generally:**	
High	4370 (87.10)
Medium	477 (9.51)
Low	170 (3.39)
**Which of the following treatments would you recommend?**	
Remineralising products and/or fluoride-based varnish or gel	3285 (65.48)
Glass-ionomer sealants	1569 (31.27)
Resin-based sealants	1960 (39.07)
Professional bleaching	393 (7.83)
Restorative treatment	3723 (74.21)
**Picture 3: dental fluorosis**	
Dental fluorosis	1430 (28.50)
Initial caries lesions	1236 (24.64)
Molar incisor hypomineralisation (MIH)	475 (9.47)
Amelogenesis imperfecta	409 (8.15)
Other	990 (19.94)
I don't know	467 (9.31)
**The caries risk in this situation is generally:**	
High	1048 (20.89)
Medium	2549 (50.81)
Low	1420 (28.30)
**Which of the following treatments would you recommend?**	
Remineralising products and/or fluoride-based varnish or gel	2913 (58.06)
Glass-ionomer sealants	1101 (21.95)
Resin-based sealants	1948 (38.83)
Professional bleaching	603 (12.02)
Restorative treatment	1652 (32.93)
**Picture 4: initial caries lesion**	
Initial caries lesion	2678 (53.38)
Dental fluorosis	843 (16.80)
Amelogenesis imperfecta	284 (5.66)
Molar incisor hypomineralisation (MIH)	235 (4.68)
Other	291 (5.80)
I don't know	686 (13.67)
**The caries risk in this situation is generally:**	
High	2130 (42.46)
Medium	2252 (44.89)
Low	635 (12.66)
**Which of the following treatments would you recommend?**	
Remineralising products and/or fluoride-based varnish or gel	3407 (67.91)
Glass-ionomer sealants	1146 (22.84)
Resin-based sealants	2027 (40.40)
Professional bleaching	526 (10.48)
Restorative treatment	2398 (47.80)

According to the logistic regression models, female dentists and younger dentists, who treated mainly children and had received information about DDEs, were more likely to recognise MIH. Dentists who correctly recognised the clinical case were more likely to classify the tooth as being at high risk of caries, recommending remineralising products and sealants with glass ionomer cements (GIC) (*P* < .01) (Table 4). Younger dentists, who treat mainly children, who have received information about DDEs, who are familiar with the characteristics of hypomineralisation and hypoplasia, and who know AI develops in the pre-eruptive phase with a genetic aetiology, were more likely to recognise AI. Dentists who correctly recognised the clinical case were more likely to classify the affected teeth at high risk of caries, recommending remineralising products, sealants with GICs and restorative therapy (*P* < .01) (Table 4). Younger dentists, who treat mainly children and are aware of what hypomineralisation is, that dental fluorosis develops in the pre-eruptive phase and can easily be confused with an initial carious lesion, were more likely to recognise it. Having received information about DDEs did not affect the odds of correctly recognising fluorosis. Dentists who correctly recognised the clinical case were more likely to classify the affected teeth as being at low risk of caries and recommend professional whitening (*P* < .01) (Table 4). Finally, dentists who were female, with more years of experience, who treated mainly children, who had received information about DDEs were more likely to recognise the ICL; those who correctly recognised the clinical case were more likely to classify the patient at high risk of caries, recommending remineralising products and resin-based sealants (Table 4).

**Table 4:** Logistics regression models.

**Picture 1: molar incisor hypomineralisation**						
	Odds ratio	Standard error	z	P>|z|	[95% conf. interval]	
Gender	1.33	0.09	4.17	0.00	1.16	1.52
Age	0.83	0.04	-4.34	0.00	0.77	0.90
Years of practice	0.93	0.04	-1.55	0.12	0.85	1.02
Patients mainly treated	0.55	0.04	-7.43	0.00	0.47	0.64
DDEs information's received	1.69	0.21	4.26	0.00	1.33	2.16
Is MIH a qualitative defect?	1.54	0.10	6.47	0.00	1.35	1.76
When does MIH develop?	1.58	0.11	6.38	0.00	1.37	1.82
Is MIH a multifactorial condition?	1.65	0.12	7.10	0.00	1.44	1.90
Is enamel hypomineralisation a qualitative defect?	1.81	0.12	8.70	0.00	1.59	2.07
*constant*	0.27	0.05	-7.32	0.00	0.19	0.38
*Log likelihood = -2956.39; Number of observations = 5.02; LR χ ^2^_(9)_ = 663.91; Prob > χ ^2^ = 0.00; Pseudo R^2^ = 0.10*						
	Odds ratio	Standard error	z	P>|z|	[95% conf. interval]	
Caries risk assessment	1.44	0.09	6.08	0.00	1.28	1.62
Remineralisation	3.53	0.31	14.54	0.00	2.98	4.18
Glass-ionomer sealants	1.91	0.13	9.75	0.00	1.68	2.17
Resin-based sealants	0.94	0.06	-0.92	0.36	0.83	1.07
Professional bleaching	0.79	0.09	-2.04	0.04	0.64	0.99
Restorative treatment	0.95	0.06	-0.72	0.47	0.84	1.08
*constant*	0.10	0.01	-19.34	0.00	0.08	0.13
*Log likelihood = -3001.39; Number of observations = 5.02; LR χ ^2^_(6)_ = 573.92; Prob > χ ^2^ = 0.00; Pseudo R^2^ = 0.09*						
**Picture 2: amelogenesis imperfecta**						
	Odds ratio	Standard error	z	P>|z|	[95% conf. interval]	
Gender	1.00	0.06	-0.04	0.97	0.88	1.13
Age	0.89	0.03	-3.03	0.00	0.82	0.96
Years of practice	0.87	0.04	-3.34	0.00	0.79	0.94
Patients mainly treated	0.81	0.06	-2.80	0.00	0.69	0.94
DDEs information's received	1.25	0.13	2.20	0.03	1.02	1.52
Is enamel hypomineralisation a qualitative defect?	1.44	0.11	4.94	0.00	1.24	1.66
Is enamel hypoplasia a quantitative defect?	1.17	0.09	2.11	0.03	1.01	1.35
When does AI develop?	1.52	0.13	4.74	0.00	1.28	1.80
Is AI a condition caused by genetic factors?	1.67	0.10	8.57	0.00	1.49	1.88
*constant*	0.78	0.13	-1.50	0.13	0.57	1.08
*Log likelihood = -3301.75; Number of observations = 5.02; LR χ ^2^_(9)_ = 345.98; Prob > χ ^2^ = 0.00; Pseudo R^2^ = 0.05*						
	Odds ratio	Standard error	z	P>|z|	[95% conf. interval]	
Caries risk assessment	2.12	0.18	9.02	0.00	1.80	2.49
Remineralisation	1.57	0.11	6.55	0.00	1.37	1.79
Glass-ionomer sealants	1.54	0.11	6.31	0.00	1.35	1.76
Resin-based sealants	0.78	0.05	-3.89	0.00	0.68	0.88
Professional bleaching	0.57	0.07	-4.78	0.00	0.45	0.72
Restorative treatment	2.31	0.16	11.69	0.00	2.00	2.65
*constant*	0.11	0.02	-13.90	0.00	0.08	0.15
*Log likelihood = -3217.70; Number of observations = 5.02; LR χ ^2^_(6)_ = 514.09; Prob > χ ^2^ = 0.00; Pseudo R^2^ = 0.07*						
**Picture 3: dental fluorosis**						
	Odds ratio	Standard error	z	P>|z|	[95% conf. interval]	
Gender	1.08	0.08	1.13	0.26	0.94	1.24
Age	0.92	0.04	-1.99	0.05	0.85	1.00
Years of practice	0.96	0.04	-0.80	0.42	0.88	1.05
Patients mainly treated	0.80	0.06	-2.74	0.01	0.68	0.94
DDEs information's received	1.09	0.12	0.73	0.47	0.87	1.35
Is enamel hypomineralisation a qualitative defect?	1.25	0.08	3.45	0.00	1.10	1.42
When does DF develop?	1.45	0.09	5.77	0.00	1.28	1.64
Do you think DF can be confused with ICL?	1.18	0.08	2.49	0.01	1.04	1.33
*constant*	0.39	0.07	-5.64	0.00	0.28	0.54
*Log likelihood = -2947.78; Number of observations = 5.02; LR χ ^2^_(8)_ = 101.18; Prob > χ ^2^ = 0.00; Pseudo R^2^ = 0.02*						
	Odds ratio	Standard error	z	P>|z|	[95% conf. interval]	
Caries risk assessment	0.37	0.02	-16.51	0.00	0.33	0.42
Remineralisation	0.22	0.02	-19.74	0.00	0.19	0.25
Glass-ionomer sealants	1.13	0.11	1.24	0.22	0.93	1.37
Resin-based sealants	1.17	0.10	1.89	0.06	0.99	1.37
Professional bleaching	4.12	0.43	13.53	0.00	3.36	5.06
Restorative treatment	1.01	0.09	0.06	0.95	0.85	1.19
*constant*	1.40	0.09	5.41	0.00	1.24	1.58
*Log likelihood = -2341.65; Number of observations = 5.02; LR χ ^2^_(6)_ = 1313.43; Prob > χ ^2^ = 0.00; Pseudo R^2^ = 0.22*						
**Picture 4: initial caries lesion**						
	Odds ratio	Standard error	z	P>|z|	[95% conf. interval]	
Gender	1.15	0.07	2.19	0.03	1.01	1.30
Age	0.97	0.04	-0.76	0.45	0.90	1.05
Years of practice	1.16	0.05	3.58	0.00	1.07	1.26
Patients mainly treated	0.53	0.04	-8.13	0.00	0.45	0.62
DDEs information's received	2.07	0.21	7.25	0.00	1.70	2.52
Do you think DF can be confused with ICL?	1.01	0.06	0.12	0.90	0.90	1.13
*constant*	0.75	0.11	-1.97	0.05	0.56	1.00
*Log likelihood = -3376.77; Number of observations = 5.02; LR χ ^2^_(6)_ = 178.56; Prob > χ ^2^ = 0.00; Pseudo R^2^ = 0.03*						
	Odds ratio	Standard error	z	P>|z|	[95% conf. interval]	
Caries risk assessment	2.88	0.16	18.76	0.00	2.58	3.21
Remineralisation	5.24	0.41	21.17	0.00	4.50	6.11
Glass-ionomer sealants	0.84	0.07	-2.09	0.04	0.71	0.99
Resin-based sealants	1.30	0.10	3.47	0.00	1.12	1.50
Professional bleaching	0.71	0.08	-3.14	0.00	0.57	0.88
Restorative treatment	1.12	0.08	1.61	0.11	0.98	1.29
*constant*	0.08	0.01	-26.86	0.00	0.07	0.10

Log likelihood = -2711.14; Number of observations = 5.02; LR χ *^2^*_(6)_ = 1509.83; Prob > χ *^2^* = 0.00; Pseudo R^2^ = 0.22.

## Discussion

The present survey investigated Italian dentists’ knowledge of Developmental Defects of Enamel, their ability to differentiate between clinical figures and to choose the most appropriate clinical approach. The response rate was relatively high (7.85%), as the trial questionnaire involved more subjects than other questionnaire-based studies on a similar topic.^19^, ^20^, ^21^, ^22^, ^23^

MIH was recognised by only slightly more than a third of the respondents and was often mistakenly confused with amelogenesis imperfecta; it can, therefore, be assumed that the clinical recognition of AI as a symmetrical defect is unknown to many dentists involved in the survey. Amelogenesis imperfecta was recognised by half of the sample and mainly confused with initial caries lesions and dental fluorosis, confirming that many dentists still need clear parameters for DDEs diagnosis. Finally, dental fluorosis was correctly identified only by just over a quarter of the sample and was mainly confused with initial caries lesions. This result is not surprising as the 2 lesions have similarities in appearance; however, the symmetry and location of the defects shown should have provided useful indications for diagnosis.

Several demographic factors were found to be associated with the correct recognition of different defects; being female, younger, and treating children were found to be positively related to accurate recognition. This finding aligns with previous research, highlighting the influence of demographic factors on diagnostic ability.^19^^,^^20^^,^^23^ MIH was identified by respondents who had reported specific training on DDEs, whereas identification was not correlated with years of work experience. This result confirmed how paediatric dentists were more able to identify MIH than general practitioners.^19^^,^^20^^,^^23^ This finding was expected and can be explained by the high percentage of children with MIH in Italy as worldwide.^30^ Due to the frequent and rapid destruction of affected teeth, general dentists may be misled into a late diagnosis, mistaking the developmental defect for a caries and thus arriving at an incorrect diagnosis. These aspects confirm the need, as expressed by general dentists, for specific training on MIH. Artificial intelligence has been described as a new method that can be adopted to increase dentists’ capabilities in identifying DDEs.^31^ Through clinical images of the defect, clinicians can be guided in diagnosing specific dental pathologies when they doubt which enamel defect they are facing. A convolutional neural network (CNN), based on learning and trained for automatic detection and categorisation of teeth with MIH on intraoral photographs, has shown higher than 95% accuracy in distinguishing caries lesions from defects due to hypomineralisation.^32^

Dentists who had not received training on DDEs are more likely to use resin than GICs to treat MIH. Different results were reported in surveys of dentists working in Northern Europe, in which a high percentage of the respondents were able to identify MIH, irrespective of the type of patients predominantly treated,^24^ and GIC was, for most respondents, the material of choice for severely affected enamel.^33^^,^^34^

Comparing the performance of dentists with that of students, as reported in a previous study in which the same clinical images were shown,^18^ reveals that both participants had difficulty recognising Dental Fluorosis and, surprisingly, the initial carious lesion, with low percentages of correct diagnoses in both cases. Amelogenesis imperfecta and MIH recognition was higher among dental students compared to practitioners. The higher recognition rates among students indicate that learning strategies are an excellent way to increase awareness and facilitate the identification of DDEs and how professional experience needs to be supported by continuous education. More comprehensive and targeted teaching methods on DDEs could raise awareness and help those already practicing the profession to recognise the broad spectrum of DDEs encountered in everyday dental practice.

In the present questionnaire submitted to dentists, as far as dental fluorosis is concerned, almost half of the sample believes it can be misdiagnosed with initial caries lesions, as was the case in the present survey and found in a previous study.^35^ This result is not surprising considering that the unexpectedly high prevalence of dental fluorosis in the population as well as in individuals reported in the literature does not match with a positive history of chronic fluoride ingestion, highlighting the need for a more precise definition and diagnosis of this condition among practitioners.^36^^,^^37^ Moreover, the prevalence of fluorosis in Italy is very low, except in confined volcanic areas. Therefore, in their work routine, Italian dentists are not used to seeing patients with these manifestations.^38^

Even amelogenesis imperfecta was not identified by almost half of the Italian dentists involved in this study; this finding confirms what other authors already stated: the late/incorrect diagnosis is still prevalent, and guidelines for treating this enamel disturbance are needed.^39^

Dentists with better diagnostic skills tend to provide more tailored and effective treatment options, which often include more conservative approaches. Indeed, when a DDE is correctly diagnosed and an intervention is needed, the clinical procedures for preventive or surgical treatments do not differ from those performed for caries management. As a result, dentists are not required to learn new procedures but to modify those already used, for example, treating hypomineralised enamel with sodium hypochlorite to improve the adhesion of the restorative material.^40^

One possible limitation of this survey might be the terms chosen to describe enamel defects in the questionnaire. Although “qualitative defects” and “quantitative defects” or Developmental Defects of Enamel are commonly used terms by academics and students, this may not be the case for general dentists not experienced in this field, which may be more accustomed to a more descriptive way of defining enamel defects, such as describing colour or extent. In addition, all the subjects included in the survey were dentists graduated in Italy. Although this factor allowed the sample to be homogeneous, it did not consent to collect data on who graduated in other countries. Finally, future investigations could be improved by investigating the training courses conducted on the topic in greater detail and presenting more clinical images to limit biases due to image interpretation. On the other hand, to the authors’ knowledge, this is the first study to investigate dentists’ knowledge of the different DDEs, their ability to distinguish the various clinical forms and to explore which treatments they would implement in individual cases, thus giving a broad overview of the topic. Moreover, the sample of dentists who answered the questionnaire is rather large and can, therefore, provide the reader with a picture of the actual abilities of Italian dentists to diagnose and treat the different DDEs. The questionnaire allows the gathering of valuable information that can be used to improve the training of future dentists and the updating for those who have only partly and distantly dealt with this subject during university studies.^41^

## Conclusion

The correct identification and management of the different types of Development Defects of Enamel is essential to provide the best possible care to patients. Within the limits of this study, Italian dentists have many areas for improvement regarding DDEs, being unable in many cases to differentiate between the different types of defects, which often leads to choosing an unsuitable treatment approach. It also shows that those who had received training on DDEs were more capable of correctly identifying the type of defect proposed and more inclined to a non-invasive approach. Despite these limitations, most of the sample correctly attributed an increased caries risk to teeth with MIH and AI. Increasing university courses and continuing education on diagnosing and managing DDEs seems reasonable to fill this knowledge gap.

## Conflict of interest

None disclosed.

## Appendix Supplementary materials

Image, application 1
