# Supplementary material for: Is Italian Dentists’ Knowledge of Enamel Development Defects Adequate? A Nationwide Survey
Source: Int Dent J. 2024 Apr 28;74(6):1447–55. doi: 10.1016/j.identj.2024.04.013 (PMC11551573; doi:10.1016/j.identj.2024.04.013)
Supplement: Supplementary file 1 [file mmc1.docx]

Supplementary files

| File | Page |
| --- | --- |
| File S1: Questionnaire | 2 |
| File S2: Content Validity Index (CVI) and the Content Validity Ratio (CVR) | 5 |
| File S3: Variables re-coding in numerical variables | 8 |

File S1.Questionnaire

| **Category** | **Questions** |
| --- | --- |
| **Demographics** | 1. **Gender** |
|  | Male |
|  | Female |
|  | 1. **Age** |
|  | ≤ 30 years |
|  | 31-40 years |
|  | 41-50 years |
|  | 51-60 years |
|  | ≥ 60 years |
|  | 1. **How many years have you been practising?** |
|  | 1-5 |
|  | 6-10 |
|  | 11-15 |
|  | ≥ 16 |
|  | 1. **Which patients do you mainly treat?** |
|  | Children (≤ 14 years) |
|  | Adults |
|  | Olders (≥ 65 years) |
| **Basic knowledge** | 1. **Have you received information regarding DDEs?** |
|  | No |
|  | Yes |
|  | 1. **If yes, where did you get information on DDEs?** |
|  | University lessons |
|  | Congresses or extra-university courses |
|  | Books |
|  | Internet |
|  | Other sources |
|  | I did not receive any information on DDEs |
|  | 1. **Enamel hypomineralization is a …… defect:** |
|  | Qualitative |
|  | Quantitative |
|  | Both |
|  | I don’t know |
|  | 1. **Enamel hypoplasia is a ……. defect:** |
|  | Qualitative |
|  | Quantitative |
|  | Both |
|  | I don’t know |
|  | 1. **Molar Incisor Hypomineralization (MIH) is a …… defect:** |
|  | Qualitative |
|  | Quantitative |
|  | Both |
|  | I don’t know |
|  | 1. **When does Molar Incisor Hypomineralization develop?** |
|  | In the pre-eruptive phase |
|  | In the post-eruptive phase |
|  | Any age |
|  | I don’t know |
|  | 1. **Molar Incisor Hypomineralization (MIH) is a condition caused by:** |
|  | Multifactorial |
|  | Genetic factors |
|  | Systemic factors |
|  | Viral or bacterial infection |
|  | I don’t know |
|  | 1. **When does Amelogenesis Imperfecta develop?** |
|  | In the pre-eruptive phase |
|  | In the post-eruptive phase |
|  | Any age |
|  | I don’t know |
|  | 1. **Amelogenesis Imperfecta is a condition caused by:** |
|  | Multifactorial |
|  | Genetic factors |
|  | Systemic factors |
|  | Viral or bacterial infection |
|  | I don’t know |
|  | 1. **When does Dental Fluorosis develop?** |
|  | In the pre-eruptive phase |
|  | In the post-eruptive phase |
|  | Any age |
|  | I don’t know |
|  | 1. **Do you think fluorosis can be confused with plaque demineralisation (Initial Caries Lesion)?** |
|  | Yes |
|  | No |
|  | I don’t know |
| **Clinical picture** | 1. **CASE 1: How would you define this condition?** |
|  | Molar Incisor Hypomineralization (MIH) |
|  | Amelogenesis Imperfecta |
|  | Dental Fluorosis |
|  | Initial Caries Lesion |
|  | Other |
|  | I don’t know |
|  | 1. **CASE 1:**  **The caries risk in this situation is generally:** |
|  | High |
|  | Medium |
|  | Low |
|  | 1. **CASE 1: Which of the following treatment would you recommend? (More answers possible)** |
|  | Remineralizing products and/or fluoride-based varnish or gel |
|  | Glass-ionomer sealants |
|  | Resin based sealants |
|  | Professional bleaching |
|  | Restorative treatment |
|  | 1. **CASE 2: How would you define this condition?** |
|  | Molar Incisor Hypomineralization (MIH) |
|  | Amelogenesis Imperfecta |
|  | Dental Fluorosis |
|  | Initial Caries Lesion |
|  | Other |
|  | I don’t know |
|  | 1. **CASE 2:**  **The caries risk in this situation is generally:** |
|  | High |
|  | Medium |
|  | Low |
|  | 1. **CASE 2: Which of the following treatment would you recommend? (More answers possible)** |
|  | Remineralizing products and/or fluoride-based varnish or gel |
|  | Glass-ionomer sealants |
|  | Resin based sealants |
|  | Professional bleaching |
|  | Restorative treatment |
|  | 1. **CASE 3: How would you define this condition?** |
|  | Molar Incisor Hypomineralization (MIH) |
|  | Amelogenesis Imperfecta |
|  | Dental Fluorosis |
|  | Initial Caries Lesion |
|  | Other |
|  | I don’t know |
|  | 1. **CASE 3:**  **The caries risk in this situation is generally:** |
|  | High |
|  | Medium |
|  | Low |
|  | 1. **CASE 3: Which of the following treatment would you recommend? (More answers possible)** |
|  | Remineralizing products and/or fluoride-based varnish or gel |
|  | Glass-ionomer sealants |
|  | Resin based sealants |
|  | Professional bleaching |
|  | Restorative treatment |
|  | 1. **CASE 4: How would you define this condition?** |
|  | Molar Incisor Hypomineralization (MIH) |
|  | Amelogenesis Imperfecta |
|  | Dental Fluorosis |
|  | Initial Caries Lesion |
|  | Other |
|  | I don’t know |
|  | 1. **CASE 4:**  **The caries risk in this situation is generally:** |
|  | High |
|  | Medium |
|  | Low |
|  | 1. **CASE 4: Which of the following treatment would you recommend? (More answers possible)** |
|  | Remineralizing products and/or fluoride-based varnish or gel |
|  | Glass-ionomer sealants |
|  | Resin based sealants |
|  | Professional bleaching |
|  | Restorative treatment |

File S2. Content Validity Index (CVI) and the Content Validity Ratio (CVR)

| **Questions’ item** | **CVR** | **Relevancy** | **Clarity** | **CVI** |
| --- | --- | --- | --- | --- |
| 1. **Gender** |  |  |  |  |
| Male | 0,67 | 1.00 | 1.00 | 1.00 |
| Female | 0,67 | 1.00 | 1.00 | 1.00 |
| 1. **Age** |  |  |  |  |
| ≤ 30 years | 1.00 | 1.00 | 1.00 | 1.00 |
| 31-40 years | 1.00 | 1.00 | 1.00 | 1.00 |
| 41-50 years | 1.00 | 1.00 | 1.00 | 1.00 |
| 51-60 years | 1.00 | 1.00 | 1.00 | 1.00 |
| ≥ 60 years | 1.00 | 1.00 | 1.00 | 1.00 |
| 1. **How many years have you been practising?** |  |  |  |  |
| 1-5 | 1.00 | 1.00 | 1.00 | 1.00 |
| 6-10 | 1.00 | 1.00 | 1.00 | 1.00 |
| 11-15 | 1.00 | 1.00 | 1.00 | 1.00 |
| ≥ 16 | 1.00 | 1.00 | 1.00 | 1.00 |
| 1. **Which patients do you mainly treat?** |  |  |  |  |
| Children (≤ 14 years) | 1.00 | 1.00 | 1.00 | 1.00 |
| Adults | 1.00 | 1.00 | 1.00 | 1.00 |
| Olders (≥ 65 years) | 1.00 | 1.00 | 1.00 | 1.00 |
| 1. **Have you received information regarding DDEs?** |  |  |  |  |
| No | 1.00 | 1.00 | 1.00 | 1.00 |
| Yes | 1.00 | 1.00 | 1.00 | 1.00 |
| 1. **If yes, where did you get information on DDEs?** |  |  |  |  |
| University lessons | 1.00 | 1.00 | 1.00 | 1.00 |
| Congresses or extra-university courses | 1.00 | 1.00 | 1.00 | 1.00 |
| Books | 1.00 | 1.00 | 1.00 | 1.00 |
| Internet | 1.00 | 1.00 | 1.00 | 1.00 |
| Other sources | 1.00 | 1.00 | 1.00 | 1.00 |
| I did not receive any information on DDEs | 1.00 | 1.00 | 1.00 | 1.00 |
| 1. **Enamel hypomineralization is a …… defect:** |  |  |  |  |
| Qualitative | 1.00 | 1.00 | 1.00 | 1.00 |
| Quantitative | 1.00 | 1.00 | 1.00 | 1.00 |
| Both | 1.00 | 1.00 | 1.00 | 1.00 |
| I don’t know | 1.00 | 1.00 | 1.00 | 1.00 |
| 1. **Enamel hypoplasia is a ……. defect:** |  |  |  |  |
| Qualitative | 1.00 | 1.00 | 1.00 | 1.00 |
| Quantitative | 1.00 | 1.00 | 1.00 | 1.00 |
| Both | 1.00 | 1.00 | 1.00 | 1.00 |
| I don’t know | 1.00 | 1.00 | 1.00 | 1.00 |
| 1. **Molar Incisor Hypomineralization (MIH) is a …… defect:** |  |  |  |  |
| Qualitative | 1.00 | 1.00 | 1.00 | 1.00 |
| Quantitative | 1.00 | 1.00 | 1.00 | 1.00 |
| Both | 1.00 | 1.00 | 1.00 | 1.00 |
| I don’t know | 1.00 | 1.00 | 1.00 | 1.00 |
| 1. **When does Molar Incisor Hypomineralization develop?** |  |  |  |  |
| In the pre-eruptive phase | 1.00 | 1.00 | 1.00 | 1.00 |
| In the post-eruptive phase | 1.00 | 1.00 | 1.00 | 1.00 |
| Any age | 1.00 | 1.00 | 1.00 | 1.00 |
| I don’t know | 1.00 | 1.00 | 1.00 | 1.00 |
| 1. **Molar Incisor Hypomineralization (MIH) is a condition caused by:** |  |  |  |  |
| Multifactorial | 1.00 | 1.00 | 1.00 | 1.00 |
| Genetic factors | 1.00 | 1.00 | 1.00 | 1.00 |
| Systemic factors | 1.00 | 1.00 | 1.00 | 1.00 |
| Viral or bacterial infection | 1.00 | 1.00 | 1.00 | 1.00 |
| I don’t know | 1.00 | 1.00 | 1.00 | 1.00 |
| 1. **When does Amelogenesis Imperfecta develop?** |  |  |  |  |
| In the pre-eruptive phase | 1.00 | 1.00 | 1.00 | 1.00 |
| In the post-eruptive phase | 1.00 | 1.00 | 1.00 | 1.00 |
| Any age | 1.00 | 1.00 | 1.00 | 1.00 |
| I don’t know | 1.00 | 1.00 | 1.00 | 1.00 |
| 1. **Amelogenesis Imperfecta is a condition caused by:** |  |  |  |  |
| Multifactorial | 1.00 | 1.00 | 1.00 | 1.00 |
| Genetic factors | 1.00 | 1.00 | 1.00 | 1.00 |
| Systemic factors | 1.00 | 1.00 | 1.00 | 1.00 |
| Viral or bacterial infection | 1.00 | 1.00 | 1.00 | 1.00 |
| I don’t know | 1.00 | 1.00 | 1.00 | 1.00 |
| 1. **When does Dental Fluorosis develop?** |  |  |  |  |
| In the pre-eruptive phase | 1.00 | 1.00 | 1.00 | 1.00 |
| In the post-eruptive phase | 1.00 | 1.00 | 1.00 | 1.00 |
| Any age | 1.00 | 1.00 | 1.00 | 1.00 |
| I don’t know | 1.00 | 1.00 | 1.00 | 1.00 |
| 1. **Do you think fluorosis can be confused with plaque demineralisation (Initial Caries Lesion)?** |  |  |  |  |
| Yes | 1.00 | 1.00 | 1.00 | 1.00 |
| No | 1.00 | 1.00 | 1.00 | 1.00 |
| I don’t know | 0,67 | 0,67 | 0,67 | 0,67 |
| 1. **CASE 1: How would you define this condition?** |  |  |  |  |
| Molar Incisor Hypomineralization (MIH) | 1.00 | 1.00 | 1.00 | 1.00 |
| Amelogenesis Imperfecta | 1.00 | 1.00 | 1.00 | 1.00 |
| Dental Fluorosis | 1.00 | 1.00 | 1.00 | 1.00 |
| Initial Caries Lesion | 1.00 | 1.00 | 1.00 | 1.00 |
| Other | 1.00 | 1.00 | 1.00 | 1.00 |
| I don’t know | 1.00 | 1.00 | 1.00 | 1.00 |
| 1. **CASE 1: The caries risk in this situation is generally:** |  |  |  |  |
| High | 1.00 | 1.00 | 1.00 | 1.00 |
| Medium | 1.00 | 1.00 | 1.00 | 1.00 |
| Low | 1.00 | 1.00 | 1.00 | 1.00 |
| 1. **CASE 1: Which of the following treatment would you recommend? (More answers possible)** |  |  |  |  |
| Remineralizing products and/or fluoride-based varnish or gel | 1.00 | 1.00 | 1.00 | 1.00 |
| Glass-ionomer sealants | 1.00 | 1.00 | 1.00 | 1.00 |
| Resin based sealants | 1.00 | 1.00 | 1.00 | 1.00 |
| Professional bleaching | 1.00 | 1.00 | 1.00 | 1.00 |
| Restorative treatment | 1.00 | 1.00 | 1.00 | 1.00 |
| 1. **CASE 2: How would you define this condition?** |  |  |  |  |
| Molar Incisor Hypomineralization (MIH) | 1.00 | 1.00 | 1.00 | 1.00 |
| Amelogenesis Imperfecta | 1.00 | 1.00 | 1.00 | 1.00 |
| Dental Fluorosis | 1.00 | 1.00 | 1.00 | 1.00 |
| Initial Caries Lesion | 1.00 | 1.00 | 1.00 | 1.00 |
| Other | 1.00 | 1.00 | 1.00 | 1.00 |
| I don’t know | 1.00 | 1.00 | 1.00 | 1.00 |
| 1. **CASE 2: The caries risk in this situation is generally:** |  |  |  |  |
| High | 1.00 | 1.00 | 1.00 | 1.00 |
| Medium | 1.00 | 1.00 | 1.00 | 1.00 |
| Low | 1.00 | 1.00 | 1.00 | 1.00 |
| 1. **CASE 2: Which of the following treatment would you recommend? (More answers possible)** |  |  |  |  |
| Remineralizing products and/or fluoride-based varnish or gel | 1.00 | 1.00 | 1.00 | 1.00 |
| Glass-ionomer sealants | 1.00 | 1.00 | 1.00 | 1.00 |
| Resin based sealants | 1.00 | 1.00 | 1.00 | 1.00 |
| Professional bleaching | 1.00 | 1.00 | 1.00 | 1.00 |
| Restorative treatment | 1.00 | 1.00 | 1.00 | 1.00 |
| 1. **CASE 3: How would you define this condition?** |  |  |  |  |
| Molar Incisor Hypomineralization (MIH) | 1.00 | 1.00 | 1.00 | 1.00 |
| Amelogenesis Imperfecta | 1.00 | 1.00 | 1.00 | 1.00 |
| Dental Fluorosis | 1.00 | 1.00 | 1.00 | 1.00 |
| Initial Caries Lesion | 1.00 | 1.00 | 1.00 | 1.00 |
| Other | 1.00 | 1.00 | 1.00 | 1.00 |
| I don’t know | 1.00 | 1.00 | 1.00 | 1.00 |
| 1. **CASE 3: The caries risk in this situation is generally:** |  |  |  |  |
| High | 1.00 | 1.00 | 1.00 | 1.00 |
| Medium | 1.00 | 1.00 | 1.00 | 1.00 |
| Low | 1.00 | 1.00 | 1.00 | 1.00 |
| 1. **CASE 3: Which of the following treatment would you recommend? (More answers possible)** |  |  |  |  |
| Remineralizing products and/or fluoride-based varnish or gel | 1.00 | 1.00 | 1.00 | 1.00 |
| Glass-ionomer sealants | 1.00 | 1.00 | 1.00 | 1.00 |
| Resin based sealants | 1.00 | 1.00 | 1.00 | 1.00 |
| Professional bleaching | 1.00 | 1.00 | 1.00 | 1.00 |
| Restorative treatment | 1.00 | 1.00 | 1.00 | 1.00 |
| 1. **CASE 4: How would you define this condition?** |  |  |  |  |
| Molar Incisor Hypomineralization (MIH) | 1.00 | 1.00 | 1.00 | 1.00 |
| Amelogenesis Imperfecta | 1.00 | 1.00 | 1.00 | 1.00 |
| Dental Fluorosis | 1.00 | 1.00 | 1.00 | 1.00 |
| Initial Caries Lesion | 1.00 | 1.00 | 1.00 | 1.00 |
| Other | 1.00 | 1.00 | 1.00 | 1.00 |
| I don’t know | 1.00 | 1.00 | 1.00 | 1.00 |
| 1. **CASE 4: The caries risk in this situation is generally:** |  |  |  |  |
| High | 1.00 | 1.00 | 1.00 | 1.00 |
| Medium | 1.00 | 1.00 | 1.00 | 1.00 |
| Low | 1.00 | 1.00 | 1.00 | 1.00 |
| 1. **CASE 4: Which of the following treatment would you recommend? (More answers possible)** |  |  |  |  |
| Remineralizing products and/or fluoride-based varnish or gel | 1.00 | 1.00 | 1.00 | 1.00 |
| Glass-ionomer sealants | 1.00 | 1.00 | 1.00 | 1.00 |
| Resin based sealants | 1.00 | 1.00 | 1.00 | 1.00 |
| Professional bleaching | 1.00 | 1.00 | 1.00 | 1.00 |
| Restorative treatment | 1.00 | 1.00 | 1.00 | 1.00 |
| **Total** | **0.99** | **1.00** | **1.00** | **1.00** |

File S3: Variables re-coding in numerical variables

| **Category** | **Questions** | **Code** |
| --- | --- | --- |
| **Demographics** | 1. **Gender** |  |
|  | Male | 0 |
|  | Female | 1 |
|  | 1. **Age** |  |
|  | ≤ 30 years | 0 |
|  | 31-40 years | 1 |
|  | 41-50 years | 2 |
|  | 51-60 years | 3 |
|  | ≥ 60 years | 4 |
|  | 1. **How many years have you been practising?** |  |
|  | 1-5 | 0 |
|  | 6-10 | 1 |
|  | 11-15 | 2 |
|  | ≥ 16 | 3 |
|  | 1. **Which patients do you mainly treat?** |  |
|  | Children (≤ 14 years) | 0 |
|  | Adults | 1 |
|  | Olders (≥ 65 years) | 2 |
| **Basic knowledge** | 1. **Have you received information regarding DDEs?** |  |
|  | No | 0 |
|  | Yes | 1 |
|  | 1. **Enamel hypomineralization is a …… defect:** |  |
|  | Qualitative | 1 |
|  | Quantitative | 0 |
|  | Both | 0 |
|  | I don’t know | 0 |
|  | 1. **Enamel hypoplasia is a ……. defect:** |  |
|  | Qualitative | 0 |
|  | Quantitative | 1 |
|  | Both | 0 |
|  | I don’t know | 0 |
|  | 1. **Molar Incisor Hypomineralization (MIH) is a …… defect:** |  |
|  | Qualitative | 1 |
|  | Quantitative | 0 |
|  | Both | 0 |
|  | I don’t know | 0 |
|  | 1. **When does Molar Incisor Hypomineralization develop?** |  |
|  | In the pre-eruptive phase | 1 |
|  | In the post-eruptive phase | 0 |
|  | Any age | 0 |
|  | I don’t know | 0 |
|  | 1. **Molar Incisor Hypomineralization (MIH) is a condition caused by:** |  |
|  | Multifactorial | 1 |
|  | Genetic factors | 0 |
|  | Systemic factors | 0 |
|  | Viral or bacterial infection | 0 |
|  | I don’t know | 0 |
|  | 1. **When does Amelogenesis Imperfecta develop?** |  |
|  | In the pre-eruptive phase | 1 |
|  | In the post-eruptive phase | 0 |
|  | Any age | 0 |
|  | I don’t know | 0 |
|  | 1. **Amelogenesis Imperfecta is a condition caused by:** |  |
|  | Multifactorial | 0 |
|  | Genetic factors | 1 |
|  | Systemic factors | 0 |
|  | Viral or bacterial infection | 0 |
|  | I don’t know | 0 |
|  | 1. **When does Dental Fluorosis develop?** |  |
|  | In the pre-eruptive phase | 1 |
|  | In the post-eruptive phase | 0 |
|  | Any age | 0 |
|  | I don’t know | 0 |
|  | 1. **Do you think fluorosis can be confused with plaque demineralisation (** **Initial Caries Lesion )?** |  |
|  | Yes | 1 |
|  | No | 0 |
|  | I don’t know | 0 |
| **Clinical picture** | 1. **CASE 1: How would you define this condition?** |  |
|  | Molar Incisor Hypomineralization (MIH) | 1 |
|  | Amelogenesis Imperfecta | 0 |
|  | Dental Fluorosis | 0 |
|  | Initial Caries Lesion | 0 |
|  | Other | 0 |
|  | I don’t know | 0 |
|  | 1. **CASE 1: The caries risk in this situation is generally:** |  |
|  | High | 2 |
|  | Medium | 1 |
|  | Low | 0 |
|  | 1. **CASE 1: Which of the following treatment would you recommend? (More answers possible)** |  |
|  | Remineralizing products and/or fluoride-based varnish or gel | Yes=1 |
|  | Glass-ionomer sealants | No=0 |
|  | Resin based sealants |  |
|  | Professional bleaching |  |
|  | Restorative treatment |  |
|  | 1. **CASE 2: How would you define this condition?** |  |
|  | Molar Incisor Hypomineralization (MIH) | 0 |
|  | Amelogenesis Imperfecta | 1 |
|  | Dental Fluorosis | 0 |
|  | Initial Caries Lesion | 0 |
|  | Other | 0 |
|  | I don’t know | 0 |
|  | 1. **CASE 2: The caries risk in this situation is generally:** |  |
|  | High | 2 |
|  | Medium | 1 |
|  | Low | 0 |
|  | 1. **CASE 2: Which of the following treatment would you recommend? (More answers possible)** |  |
|  | Remineralizing products and/or fluoride-based varnish or gel | Yes=1 |
|  | Glass-ionomer sealants | No=0 |
|  | Resin based sealants |  |
|  | Professional bleaching |  |
|  | Restorative treatment |  |
|  | 1. **CASE 3: How would you define this condition?** |  |
|  | Molar Incisor Hypomineralization (MIH) | 0 |
|  | Amelogenesis Imperfecta | 0 |
|  | Dental Fluorosis | 1 |
|  | Initial Caries Lesion | 0 |
|  | Other | 0 |
|  | I don’t know | 0 |
|  | 1. **CASE 3: The caries risk in this situation is generally:** | Multiple-chiose |
|  | High | 2 |
|  | Medium | 1 |
|  | Low | 0 |
|  | 1. **CASE 3: Which of the following treatment would you recommend? (More answers possible)** |  |
|  | Remineralizing products and/or fluoride-based varnish or gel | Yes=1 |
|  | Glass-ionomer sealants | No=0 |
|  | Resin based sealants |  |
|  | Professional bleaching |  |
|  | Restorative treatment |  |
|  | 1. **CASE 4: How would you define this condition?** |  |
|  | Molar Incisor Hypomineralization (MIH) | 0 |
|  | Amelogenesis Imperfecta | 0 |
|  | Dental Fluorosis | 0 |
|  | Initial Caries Lesion | 1 |
|  | Other | 0 |
|  | I don’t know | 0 |
|  | 1. **CASE 4: The caries risk in this situation is generally:** |  |
|  | High | 2 |
|  | Medium | 1 |
|  | Low | 0 |
|  | 1. **CASE 4: Which of the following treatment would you recommend? (More answers possible)** |  |
|  | Remineralizing products and/or fluoride-based varnish or gel |  |
|  | Glass-ionomer sealants | Yes=1 |
|  | Resin based sealants | No=0 |
|  | Professional bleaching |  |
|  | Restorative treatment |  |
